# Anatomical Variations of the Bifurcation Levels of the Common Carotid Artery and Superior Thyroid Artery

**DOI:** 10.7759/cureus.71120

**Published:** 2024-10-09

**Authors:** Mahil Abdalla, Najla Mohammed, Ragda Abdallah, Marwan K Ahmed, Muaaz Ismaiel, Mohanad Abdelrahim, Abrar Salih, Eram Yousif, Abdelmohaymin A Abdalla, Mohamed A Abdelrahim

**Affiliations:** 1 Physiology, Neuroscience, and Behavioral Sciences, St. George's University School of Medicine, True Blue, GRD; 2 Neurological Surgery, National Centre of Neurological Sciences, Khartoum, SDN; 3 Anatomical Sciences, St. George's University School of Medicine, True Blue, GRD; 4 Internal Medicine, Ascension Saint Joseph, Chicago, USA; 5 Internal Medicine, Faculty of Medicine, University of Khartoum, Khartoum, SDN; 6 Internal Medicine, Mersey and West Lancashire Teaching Hospitals NHS Trust, Prescot, GBR; 7 Public Health, Al Neelain University, Khartoum, SDN; 8 Pulmonary and Critical Care Medicine, Mayo Clinic Hospital, Phoenix, USA

**Keywords:** atherosclerosis, cca bifurcation, common carotid artery, embalmed cadavers, external carotid artery, sudanese, superior thyroid artery

## Abstract

Background: Variations in the carotid arterial system significantly impact atherosclerosis's clinical and surgical management, which predominantly occurs at arterial bifurcations.

Objectives: This study aimed at evaluating anatomical variations in the bifurcation levels of the common carotid artery (CCA) and the superior thyroid artery (STA), emphasizing clinical relevance.

Methods: This was an observational cross-sectional study conducted in June 2021 using 32 embalmed cadavers of adult age from medical schools in Khartoum, Sudan. Sixty-four carotids were examined, noting the levels of CCA bifurcation and STA origin, and their symmetry across both sides of the body.

Results: Among the cadavers studied, CCA bifurcation most commonly occurred at the superior border of the thyroid cartilage (46.9%), followed by the body of the hyoid bone (40.1%). The STA predominantly originated from the external carotid artery (ECA) (65.6%).

Conclusion: No significant correlation was found between the bifurcation levels of the CCA and the origins of the STA. Bilateral symmetry in the bifurcation of the CCA and the origin of the STA was observed in most cases. The study highlights notable variations in the anatomy of the carotid arteries. These findings suggest that pre-surgical imaging and careful planning are crucial to accommodate anatomical diversities.

## Introduction

Atherosclerosis is a widespread condition that affects thousands globally, often leading to complications, including stroke [1]. The disease predominantly arises in regions of arterial bifurcations such as the carotid artery bifurcation sites [2]. At these bifurcation sites, disruptions in local blood flow alter wall shear stress, contributing to endothelial damage and the development of atherosclerotic lesions [3]. Given that atherosclerosis in the carotid arterial system is responsible for 25% of stroke cases, a detailed understanding of carotid artery anatomy, structure, and bifurcations is essential for diagnosis and treatment strategies [4]. A thorough understanding of the carotid arterial system's anatomy is crucial for the surgical management of atherosclerotic plaques that form around the carotid bifurcation [5]. Numerous anatomical variations within the carotid arterial system have been documented, with varying prevalence observed across different populations [6]. These variations are thought to arise from the intricate process of arterial development during the embryological formation of the carotid arterial system [7]. Knowledge of the anatomy of the external, internal, and common carotid arteries is critical for radical neck surgeries to define the plane of dissection. Carotid bifurcations serve as a key surgical landmark, and hence a thorough understanding of the variations and lengths of the carotid arteries at the bifurcation sites is also essential prior to performing carotid endarterectomy [8]. Due to the asymmetrical origins of the right and left common carotid arteries, their lengths differ [9]. Generally, the carotid bifurcation level is considered symmetrical in relation to the cervical vertebrae or cartilages of the larynx [10]. Although population variations with regard to bifurcation levels have been reported, sex-based variations have not been extensively studied [11]. Surgeries involving carotid arteries are highly risky and demand precise dissection due to the intricate anatomy of the region [10, 11]. Anatomical references vary in their descriptions of the relationship between the common carotid artery (CCA) and nearby structures, as well as the different levels of bifurcation [10,12-15]. This study aims to investigate the bifurcation levels in Sudanese cadavers, given their significant clinical and surgical relevance. A thorough understanding of this anatomical structure is crucial for reducing operative risks and improving outcomes in surgeries involving the CCA [10].

## Materials and methods

This was an observational cross-sectional study conducted in June 2021, designed to observe anatomical variations in the bifurcation of the CCA and the origin of the superior thyroid artery (STA). The study involved 32 Sudanese cadavers obtained from the Faculties of Medicine at the University of Khartoum, Al Neelain University, and Napata College, Khartoum, Sudan. These cadavers were prepared for anatomical dissection courses. A total of 64 carotid arteries were examined in 32 human cadavers (two carotid arteries per cadaver).

Inclusion and exclusion criteria

All available cadavers that were properly preserved for dissection and had intact carotid arteries were included in the study.Cadavers with visible deformities, trauma, or any form of damage to the neck region or carotid arteries were excluded.

Data collection

The following parameters were measured: the level of bifurcation of the CCA, the level of origin of the STA, and the bilateral symmetry of the carotid bifurcation. Data collection was performed through direct observation of the bifurcation of the CCA and the level of origin of the STA. Bilateral symmetry was also noted. A cotton thread was used to identify the level of the carotid bifurcation to the nearby anatomical landmarks, which were the body of the thyroid cartilage, the superior border of the thyroid cartilage, greater cornu of the hyoid bone, and the body of the hyoid bone.

Sample calculation

Since this study utilized cadavers available for dissection, a sample calculation was not performed. The study was conducted on all cadavers meeting the inclusion criteria within the study period.

Statistical analysis

Data were analyzed using IBM SPSS Statistics for Windows, Version 20.0 (Released 2011; IBM Corp., Armonk, New York, United States). Descriptive statistics were performed for data cleaning and summarization. Correlation coefficients were calculated to explore associations between parameters, and P-values were computed to determine the statistical significance, with a P-value of < 0.05 considered significant.

Ethical considerations

Ethical approval for this study was granted by the National Research Health Ethics Committee (NRHEC) - Ministry of Health, Khartoum, Sudan (approval number MOH/KS/GDTM/44/A). The Anatomy Departments of Al Neelain University, the University of Khartoum, and Napata College accepted the decision of the NRHEC. The study adhered to both national and international ethical standards for the use of human cadavers in research.

## Results

The symmetry and level of bifurcation of the CCA

The sample consisted of 32 cadavers (28 males and four females), all adults (aged more than 18 years), resulting in a total of 64 carotid artery specimens (two carotids per cadaver). The CCA bifurcation was symmetrical between the right and left sides among all cadavers examined. The relationship between the CCA bifurcation and the structures of the anterior larynx was assessed across these specimens. The bifurcation site of the CCA was most frequently found at the superior border of the thyroid cartilage, accounting for 30 specimens (46.9%). This was followed by bifurcation at the body of the hyoid bone in 26 specimens (40.6%). A lesser number of specimens (n=8, 12.5%) demonstrated bifurcation at the body of the thyroid cartilage. Notably, none of the specimens showed a bifurcation at the tip of the greater horn of the hyoid bone. These distributions are summarized in Table 1.

**Table 1:** The level of CCA bifurcation (N= 64) in the cadavers used in the study CCA: common carotid artery

Level of Carotid Bifurcation	Carotid Bifurcation (Percentage)	Carotid Bifurcation (Frequency)
Superior border of the thyroid cartilage	46.9%	30
Body of the hyoid	40.6%	26
Body of the thyroid cartilage	12.5%	8
Greater horn of the hyoid	00.0%	0

Origin of the STA in the cadavers used in the study

**Figure 1:**
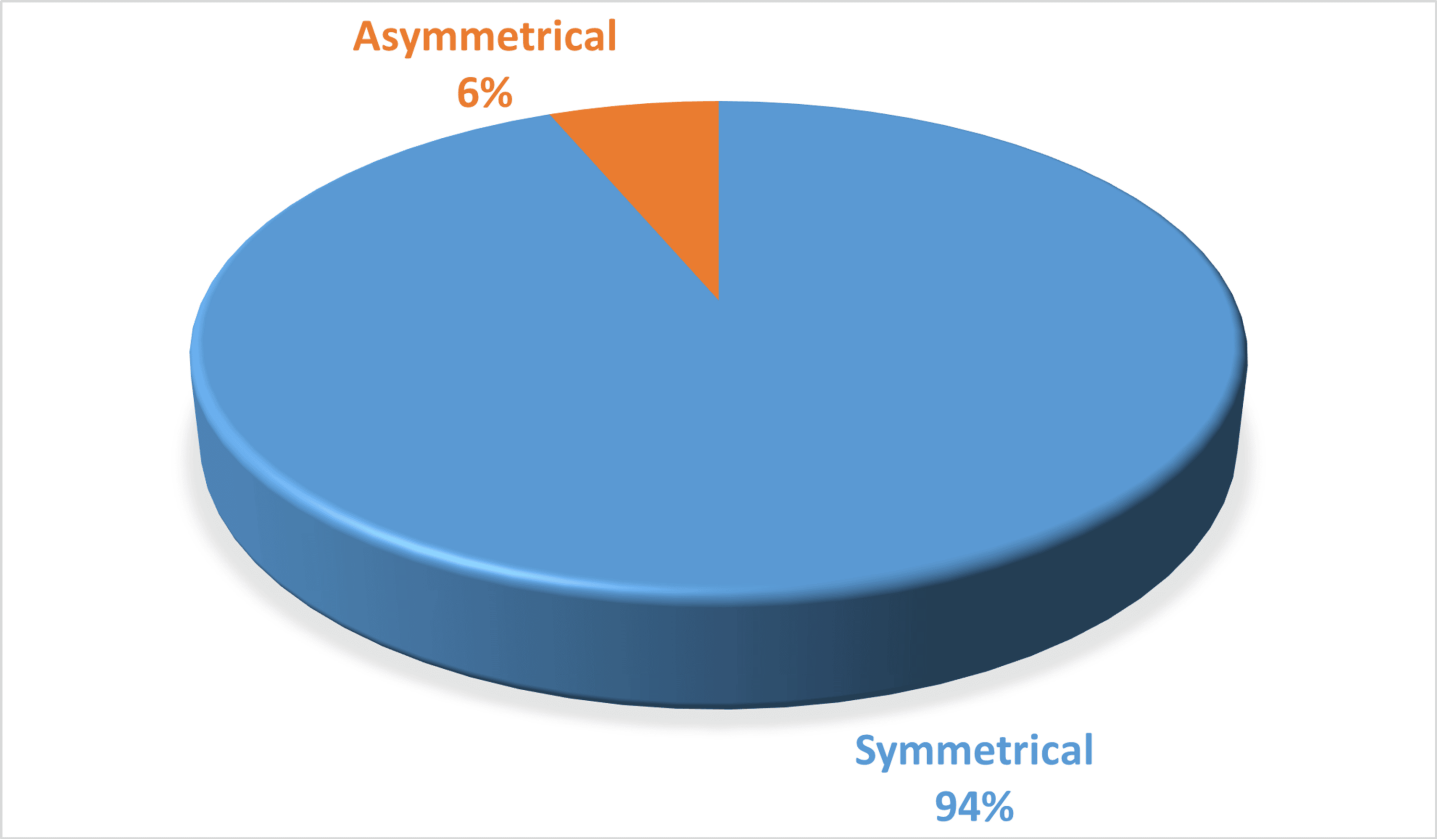
The symmetry in the origin of the STA (N=64) in the cadavers used in the study STA: superior thyroid artery

When analyzing the origin of the STA, the 64 specimens revealed that the STA predominantly originated from the external carotid artery (ECA) in 42 specimens (65.6%). In contrast, the STA originated from the CCA bifurcation in 21 specimens (32.8%), and only one specimen (1.6%) had the STA originating from the CCA itself. These findings are detailed in Table 2. Notably, the origin of the STA was asymmetrical in two of 32 cadavers (6.3%) (Figure 1).

**Table 2:** Origin of the STA (N=64) in the cadavers used in the study STA: superior thyroid artery

Origin of the STA	Percentage	Frequency
External carotid artery	65.6%	42
Carotid artery bifurcation	32.8%	21
Common carotid artery	1.6%	1

Additionally, the study examined the level of STA origin in relation to anatomical landmarks. The STA originated most commonly at the level of the greater horn of the hyoid, seen in 29 specimens (45.3%). The body of the hyoid was the second most common origin site, observed in 21 specimens (32.8%). The superior border of the thyroid cartilage was identified as the site of STA origin in 12 specimens (18.8%), while only two specimens (3.1%) showed origin at the level of the body of the thyroid cartilage. These findings are presented in Table 3.

**Table 3:** The level of origin of the STA (N= 64) in the cadavers used in the study STA: superior thyroid artery

Level of Origin of the STA	Percentage	Frequency
Greater horn of the hyoid	45.30%	29
Body of the hyoid	32.80%	21
Superior border of the thyroid cartilage	18.80%	12
Body of the thyroid cartilage	3.10%	2

Relationship between the origin of the STA and the level of CCA bifurcation

Table 4 presents the relationship between the origin of the STA and the level of the CCA bifurcation in a sample of Sudanese cadavers. The data indicate that there is no significant relation between the origin of the STA and the level of CCA bifurcation, as evidenced by p-values greater than 0.05 for all categories assessed. Specifically, when the STA originated at the level of the body of the hyoid, the distribution across the CCA, carotid artery bifurcation, and ECA were 0%, 46.2%, and 53.8%, respectively, with a p-value of 0.136. When the STA originated at the level of the superior thyroid cartilage, 3.3% were from the CCA, 23.3% from the bifurcation of the carotid artery, and 73.3% from the ECA, resulting in a p-value of 0.203. Lastly, for the STA origin at the level of the body of the thyroid cartilage, the distribution was 0% from the CCA, 25.0% from the bifurcation, and 75.0% from the ECA, with a p-value of 0.804. None of these results reach statistical significance, as all p-values are above the threshold of 0.05.

**Table 4:** Relationship between the origin of the STA and the level of the CCA bifurcation p-value < 0.05 was considered significant *Refers to 12 out of a subset of samples, not the full 64, where the bifurcation of the CCA was at the level of the body of the hyoid. The 46.2% corresponds to this subset (in this case, 26), not the full sample **Indicates 14 cases from the same subset (n-26) where the origin of the STA was at the body of the hyoid. The subset in this row appears to be 26 (12 + 14 = 26), which gives the percentages of 46.2% and 53.8%, within this specific group. NOTE: For each row, the counts and percentages represent specific subsets of the total sample size (n=64), based on the anatomical relationship being analyzed. Therefore, the percentages do not always reflect the entire sample, but rather a portion of the sample relevant to that particular anatomical relationship. CCA: common carotid artery; STA: superior thyroid artery; ECA: external carotid artery

Level of CCA Bifurcation	Origin of STA			p-value
	CCA, n (%)	Bifurcation of Carotid Artery, n (%)	ECA, n (%)	
Body of hyoid (n=26)	0 (0%)	12 (46.2%)*	14 (53.8%)**	.136
Superior thyroid cartilage (n=30)	1 (3.3%)	7 (23.3%)	22 (73.3%)	.203
Body of thyroid cartilage (n=8)	0 (0%)	2 (25.0%)	6 (75.0%)	.804

## Discussion

Most studies conclude that the CCA bifurcates at the level of the superior border of the thyroid cartilage [10,12-14,16-19]. In this study, the bifurcation of the CCA was found to be at a similar level to the superior border of the thyroid cartilage, followed by the body of the hyoid bone. The similarity between the results of this study and international findings can be attributed to the cultural diversity resulting from intermarriage within the Sudanese population. In addition, in the classic literature, the CCA bifurcation occurs at the level of the C4 vertebra or C3-C4 intervertebral level, which is the same level as the thyroid cartilage’s upper border [18,19]. In this study, there was no observed CCA bifurcation at the level of the greater horn of the hyoid bone. However, some authors have reported the CCA dividing at the upper level of the greater horn of the hyoid bone [10,15]. This discrepancy may be due to ethnic differences between African and European populations. The STA is commonly observed as a branch of the ECA, as noted in previous studies [20-22]. Consistent with these findings, our study demonstrated that in approximately two-thirds of the cadavers examined, the STA originated from the ECA. Comparable variations were reported in the Ethiopian population, where the STA arose from the ECA in 44.2% of cases and from the CCA bifurcation in 27.9% of cases [23]. Furthermore, other studies have documented cases where the STA originated from the internal carotid artery (ICA) or the subclavian artery (SCA) [23-26]. The development of the CCA and ICA from the third aortic arch, with the ECA branching cranially as a new vessel from the same arch, suggests that these variations may be attributed to alterations in the embryonic development of the aortic arch [23,27-29].

This study found that the levels of bifurcation of the CCA were symmetrical, which aligns with another cadaveric study reporting 100% symmetry in bifurcation levels [10]. However, a study using carotid angiograms reported that the right CCA bifurcation was lower than the left in nearly half of the living specimens [30]. Another study observed that in 42.5% of a population of 247 living individuals, the right bifurcation level was lower than the left, while only 23.9% exhibited bilateral symmetry [18]. The discrepancy between findings from cadaveric and live human studies warrants further investigation to better understand the underlying causes. The origin of the STA is closely related to the level of the CCA bifurcation. A higher bifurcation level of the CCA is often associated with the STA originating from the bifurcation itself, while a lower bifurcation level tends to correlate with the STA branching from the ECA. Similar findings have been reported by [23,26,28], and despite differences in the populations studied, these variations may represent normal anatomical differences rather than pathological anomalies.

In this study, 30 out of 32 cadavers had a symmetrical origin of the STA, contrasting with a study by Toni et al., which found differences in STA origins between sides of the neck among East Asians [31]. This asymmetry in STA origin may be due to the asymmetrical development of the aortic arch and thyroid bud [32], possibly due to increased vascular demand of the tissues during development [33].

Recommendations

Surgeons performing carotid endarterectomy need to be aware of the complex anatomical variations. Recognizing a high CCA bifurcation before surgery can aid in surgical planning. This study suggests that a high bifurcation increases the likelihood of the STA emerging from the CCA. Imaging investigations such as angiography and duplex ultrasound are crucial for identifying anatomical variations and minimizing collateral injuries. Clinical signs alone are not reliable for assessing the level of bifurcation. Future studies with larger and more diverse samples that specify the gender are recommended for more comprehensive results.

Limitations

The small sample size of cadavers (especially female cadavers) used in this study may limit the generalizability of the findings to the broader Sudanese population.

## Conclusions

This study found that the primary location of the CCA bifurcation was at the level of the superior border of the thyroid cartilage. The origin of the STA is related to the level of the CCA bifurcation; a high bifurcation level of the CCA is associated with the STA branching off from the CCA bifurcation site, while a lower CCA bifurcation is associated with the STA originating from the ECA. Anatomical high bifurcations should alert specialists to the possibility of the STA branching from the CCA bifurcation, or, as found in one cadaver, from the CCA itself.
